# Supplementary material for: Cohort profile: a longitudinal study of HIV infection in the central nervous system with focus on cerebrospinal fluid – the Gothenburg HIV CSF Study Cohort
Source: BMJ Open. 2023 May 17;13(5):e070693. doi: 10.1136/bmjopen-2022-070693 (PMC10193099; doi:10.1136/bmjopen-2022-070693)
Supplement: Author's manuscript [file bmjopen-2022-070693.draft_revisions.pdf]

BMJ Open is committed to open peer review. As part of this commitment we make the peer review history of every article we publish publicly available.

When an article is published we post the peer reviewers' comments and the authors' responses online. We also post the versions of the paper that were used during peer review. These are the versions that the peer review comments apply to.

The versions of the paper that follow are the versions that were submitted during the peer review process. They are not the versions of record or the final published versions. They should not be cited or distributed as the published version of this manuscript.

BMJ Open is an open access journal and the full, final, typeset and author-corrected version of record of the manuscript is available on our site with no access controls, subscription charges or pay-per-view fees (http://bmjopen.bmj.com).

If you have any questions on BMJ Open's open peer review process please email info.bmjopen@bmj.com

BMJ Open

Cohort profile: A longitudinal study of HIV infection in the central nervous system with focus on cerebrospinal fluid- The Gothenburg HIV CSF Study Cohort

Journal:	*BMJ Open*
Manuscript ID	bmjopen-2022-070693
Article Type:	Cohort profile
Date Submitted by the Author:	01-Dec-2022
Complete List of Authors:	Hagberg, Lars; University of Gothenburg, Department of Infectious Diseases<br>Gisslén, Magnus; University of Gothenburg Sahlgrenska Academy, Department of Infectious Diseases, Institute of Biomedicine; Sahlgrenska University Hospital, Department of Infectious Diseases, Region Västra Götaland
Keywords:	HIV & AIDS < INFECTIOUS DISEASES, MICROBIOLOGY, Infectious disease/HIV < NEUROLOGY

SCHOLARONE™
Manuscripts

I, the Submitting Author has the right to grant and does grant on behalf of all authors of the Work (as defined in the below author licence), an exclusive licence and/or a non-exclusive licence for contributions from authors who are: i) UK Crown employees; ii) where BMJ has agreed a CC-BY licence shall apply, and/or iii) in accordance with the terms applicable for US Federal Government officers or employees acting as part of their official duties; on a worldwide, perpetual, irrevocable, royalty-free basis to BMJ Publishing Group Ltd ("BMJ") its licensees and where the relevant Journal is co-owned by BMJ to the co-owners of the Journal, to publish the Work in this journal and any other BMJ products and to exploit all rights, as set out in our licence.

The Submitting Author accepts and understands that any supply made under these terms is made by BMJ to the Submitting Author unless you are acting as an employee on behalf of your employer or a postgraduate student of an affiliated institution which is paying any applicable article publishing charge ("APC") for Open Access articles. Where the Submitting Author wishes to make the Work available on an Open Access basis (and intends to pay the relevant APC), the terms of reuse of such Open Access shall be governed by a Creative Commons licence – details of these licences and which Creative Commons licence will apply to this Work are set out in our licence referred to above.

Other than as permitted in any relevant BMJ Author's Self Archiving Policies, I confirm this Work has not been accepted for publication elsewhere, is not being considered for publication elsewhere and does not duplicate material already published. I confirm all authors consent to publication of this Work and authorise the granting of this licence.

For peer review only

BMJ Open Cohort profile

Title: Cohort profile: A longitudinal study of HIV infection in the central nervous system with focus on cerebrospinal fluid-The Gothenburg HIV CSF Study Cohort

Authors: Lars Hagberg[1,2], Magnus Gisslén[1,2]

1. Department of Infectious Diseases, Institute of Biomedicine, Sahlgrenska Academy, University of Gothenburg, Gothenburg, Sweden
2. Region Västra Götaland, Sahlgrenska University Hospital, Gothenburg, Sweden

Corresponding author: Lars Hagberg.

Postal address: Dept of Infectious Diseases, Sahlgrenska University Hospital SE 416 85 Gothenburg, Sweden.

Email: lars.hagberg@gu.se

Word count: 3373

For peer review only

ABSTRACT

Purpose: In order to enable long-term follow-up of the natural course of HIV infection in the central nervous system (CNS), a longitudinal cohort study with repeated cerebrospinal fluid (CSF) analyses at intervals over time was initiated in 1985. When antiretrovirals against HIV were introduced, short- and long-term effects of various antiretroviral treatment (ART) regimens were added.

Participants: Adults people living with HIV (PLWH) were included. Most participants were asymptomatic which distinguishes this cohort from other similar studies. Since lumbar puncture (LP) is an invasive procedure, some PLHW withdraw their consent. Furthermore, some participants were lost to follow-up. Of 662 PLWH where an initial LP was done, 415 agreed to continue. Among the 415, 56 only gave permission to be followed with LP for less than 1 year, mainly to analyze the short-term effect of ART. The remaining 359 PLWH were followed-up with repeated LP for periods ranging from > 1 to 30 years, defined as the "longitudinal cohort". On 7 April 2022, 2,650 samplings of paired CSF/blood had been performed, providing a unique biobank.

Findings to date: A general finding was that HIV infection in the CNS, as mirrored by CSF findings, appears early in the infectious course of the disease, and progresses slowly in the vast majority of untreated PLWH. Combination ART has been highly effective in reducing CSF viral counts, inflammation, and markers of neural damage. Minor CSF signs of long-term sequels or residual inflammatory activity and CSF escape (viral CSF blips) have been observed. The clinical impact of these findings require further studies.

Future plans: PLWH today have a long life expectancy**.** Our cohort provides an unique opportunity to study long-term effects on CNS HIV infection and the impact of ART.

Keywords: HIV infection, central nervous system, cerebrospinal fluid

For peer review only

Strengths and limitations

- Difficult to follow the protocol with yearly repeated LP in several participants due to consent and Covid-19 pandemic.
- Infrequent use of extensive neuropsychiatric testing.
- Only limited number of participants suffering from severe neurological complications and opportunistic CNS infections.
- Strength of our study is its uniquely long follow-up time, with CSF data from a population of PLWH with a predominately neuroasymptomatic clinical appearance, together with full clinical history including comorbidities, treatment and laboratory data.
- A strict protocol for collecting and storing CSF/blood samples at one center, and only engaging a handful of clinicians to enhance consistency and uniformity is another strength.

For peer review only

INTRODUCTION

Chronic untreated HIV infection causes progressive immunodeficiency that leads to AIDS in a median of 10 or 11 years after a primary infection. Before effective treatment was available, HIV-associated dementia was frequently observed in the late stages of the infection. The introduction of combination antiretroviral treatment (ART), that preserves or restores immune functions has had a major impact on morbidity and mortality. It has also resulted in a marked reduction of HIV-associated dementia and other neurologic complications. (1) Nevertheless, mild forms of HIV-associated neurocognitive disorders have frequently been reported even during suppressive ART. (2) The research questions for the present study have been: Can biomarkers in cerebrospinal fluid (CSF) be used to chart the natural course of central nervous system (CNS) HIV infection, and can we determine the short and long-term effects of ART?

Cohort description and method

Study population

The study was initiated in 1985 when two patients attending our clinic presented with Guillain Barre syndrome as a complication to HIV infection and exhibited HIV isolated in the CSF. (3) Later we found that HIV-1 could also be isolated in CSF of virus carriers who had no neurological symptoms. (4) At that time, it was not known that HIV was neurotropic.

Since 1985, HIV-infected people living with HIV (PLWH) in the Gothenburg area of Sweden have been enrolled in a longitudinal study with serial sampling of CSF and blood. Lumbar punctures (LP) are performed annually, if possible, or more frequently in connection with introduction or cessation of ART. Both PLWH with neurological or other clinical symptoms of HIV, and asymptomatic PLWH have been included in the study.

For peer review only

Of 662 PLWH in whom an initial LP was done, 415 PLWH agreed to continue with follow-up. Among the 415, 56 only gave permission to be followed with LP for less than 1 year, mainly to analyze the short-term effect of ART. The remaining 359 PLWH were followed with repeated LP for periods > 1 up to 30 years. This group was defined as the "longitudinal cohort" (see flow chart, Fig.1). The 247 PLWH who participated with only one CSF/blood sampling have been included in many cross-sectional studies. At the conclusion of the study, on 7 April 2022, 2,650 LP and samplings of paired CSF/blood had been performed, providing a unique biobank.

Longitudinal cohort

In total, 359 PLWH were followed with a mean number of 6.26 (range 2–30) LP and CSF/blood analyses over a mean period of 6.89 years (range 1–30 years). Patient characteristics, time of follow-up, and number of CSF analyses is shown in Table 1.

Table 1. Longitudinal cohort characterization

Number of participants followed > 1 year: 359

Mean time of follow-up: 7 years (range 1–30)

Number of lumbar punctures/individuals: 6 (range 2–30)

Age at inclusion, mean: 41 years (range 17–73)

Gender, male/female: 247/112

Geographic background (n):

Sweden 182

Europe (outside Sweden) 37

Africa 93

Asia 35

For peer review only

Middle East 3

America 9

HIV1/HIV2 356/3

mean CD4+ cells at inclusion: 369 x10^6/L (range 0–2131)

CDC classification[1] at inclusion: A1-A3 n = 244; B1-B3 n = 34; C1-C3 (AIDS) n = 81

ART at inclusion (n): No treatment: 298 (of whom 31 had primary infection)

Treatment suppressed: 49

Treatment failure: 11

Treatment interruption: 1

Neurology at inclusion (n): Neuroasymptomatic: 328

CNS opportunist: 12

HIV-associated dementia: 10

Other CNS complications: 9

---

[1]Revised classification system for HIV: MMWR Dec 18; 41:1-19. 1993

About half of the participants (51%) were born in Sweden (n = 182) and 177 (49%) outside Sweden, most commonly in sub-Saharan Africa (n = 93) (26%), Europe outside Sweden (n = 37) (10%), and Asia (n = 35) (10%). The Covid-19 pandemic partially halted the study for a period. However, 166 PLWH (46%) had been followed for more than 5 years and 65 of these (18%) have been followed for more than 10 years, providing great opportunities for longitudinal evaluations. A total of 121 PLWH were diseased or lost to follow-up. The remaining 238 PLWH are eligible to ask for further follow-up (see flow chart Fig. 1).

In addition, 94 HIV-negative healthy controls have been recruited for CSF/blood sampling, of whom 53 are men on HIV pre-exposure prophylaxis (PrEP). Follow-up sampling of this cohort is ongoing.

For peer review only

Patient and public involvement: The local patient organization (PG vast) was regularly informed of the study and the results.

Validation of the study population

PLWH whose CSF/blood was sampled only once and declined to continue the study because of discomfort from the initial LP, or were too sick for follow-up, or were below 18 years of age, or were lost to follow-up were registered in the reject log. That log included 247 PLWH with a mean age of 40 years (range 2–76), of whom 74 (30%) were women and 173 (70%) were male. Their mean CD4 cell count was 344 x10^6/L, (range 10–1420); 40% were classified as CDC stage C1-C3 (AIDS). The number of AIDS patients (40%) were larger than the longitudinal cohort group (22%), but otherwise age, gender, and CD4 cell count was of similar magnitude between the groups.

Cohort variables and laboratory analyses

Full clinical history, including co-morbidity, treatment, and laboratory results were collected throughout the study. Variables recorded at enrolment included sex, country of birth, mode of HIV transmission, date of last HIV-negative test (if any), and first positive HIV-test, and suspected country for HIV transmission. Data collected/updated at each follow-up visit included ART, prophylaxis of opportunistic infections, and co-medications administered. Data on co-infection with hepatitis C and B virus, weight, date and type of AIDS-defining events and non-AIDS events, and date and cause of death were also included. HIV-RNA, CD4+ and CD8+ T-cell counts and CD4/CD8 ratios, HIV subtype and HIV drug resistance (including viral sequences) were also recorded.

For peer review only

CSF and blood collection took place in the morning, before breakfast in a standardized manner with the subject in a lateral recumbent position. Twenty-four mL of CSF were collected and centrifuged for cell counting. CSF cells and buffy coat were stored separately, and cell-free CSF, serum, and plasma were divided into fractions. Fractions not immediately analyzed were stored at –70°C in the local laboratory after collection. In conjunction with each LP. virological, immunological, and neuronal injury markers in the CSF were compared with the clinical course. The laboratory analyses were grouped in three categories.

1. Virology
2. Markers of inflammation and immunology
3. Markers of CNS injury

1. *Virology*: Since its introduction in 1996 quantitative HIV-1 RNA polymerase chain reaction (PCR) has been used as the main marker of viral load (currently Cobas Taqman v.2, Roche Diagnostic Systems, Hoffmann-La Roche, Inc., Basel, Switzerland). Quantitative HIV-1 DNA real-time PCR (TaqMan5´nuclease) assay has been analyzed in sub-populations. Prior to 1996, CSF HIV antigen test and virus isolation were included in the protocol.

2. *Markers of inflammation and immunology*; Neopterin concentration reflects macrophage activity and has been the main marker of CSF inflammation (RIA method Henning test Neopterin, BRAHMS, Berlin Germany). Among other procedures CSF monocyte cell count, protein electrophoresis, oligoclonal bands, and beta-2-microglobulin concentration have been analyzed in all patients. Various cytokines such as MCP-1, IP-10, CXCL10, uPA, and suPAR have also been measured in sub-populations.

For peer review only

3. *Markers of CNS injury*: CSF neurofilament light protein (Nfl) concentration that reflects axonal damage has been the main marker used to estimate CNS injury in CSF (NF-light ELISA kit; Uman Diagnostics AB, Umeå, Sweden), and ultrasensitive single molecule array (Simoa) method has been used for blood. Other markers such as gangliosides (GM1, GD1a, GD1b, and GT1b), sulfatides, glial markers, including GFAP (astroglia) and GD3 (microglial/macrophages), t-tau, p-tau, beta-amyloids, s-APP and neurogranin (synapse protein) have been studied in sub-populations.

Neuropsychiatric test

Reaction time tests have been performed in sub-populations (5) (6). Since 2011, neurocognitive testing has been done with a computerized cognitive test battery (Cogstate, Melbourne, Australia) that has been validated for HIV-infected individuals (7, 8). Four different tests from the Cogstate Brief Battery were used to assess five cognitive domains: the Detection Test measured psychomotor function and attention, the Identification test assessed speed of information processing and attention, the One Card Learning test evaluated learning, and the One Back memory test assessed working memory. (9)

RESULTS

A general finding resulting from our 37-year study is that HIV-1 infection in the CNS, as mirrored by CSF findings, appears early during the infectious course of the disease, and progresses slowly in the vast majority of untreated PLWH. (10, 11) Combination ART has been very effective in reducing CSF HIV-1 loads, inflammation, and markers of neural

For peer review only

damage. (12, 13). However, minor CSF signs of long-term sequela or residual inflammatory activity have been observed during follow-up. (14-17).

When several CSF specimens from the same individual were studied, HIV-1 could be isolated from 80% of them and detected by PCR in 90% of cases, (10) with higher HIV-RNA cutoff levels required in CSF than in blood to predict positive HIV-1 isolation. (18) The viral load was approximately (mean) 1 log lower in CSF than in blood, (19-21) but CSF viral load exceeded plasma levels (CSF > plasma discordance) in 13%, with variations between different disease stages, ranging from 1% in primary HIV, 11% in neuroasymptomatic patients, and up to 30% in patients with HIV-associated dementia. (19, 21) HIV-1 RNA levels increased in CSF relative to time of infection. (11) Markers of immune stimulation such as neopterin and beta-2 microglobulin also increased in CSF during follow-up, indicating that HIV-1 CNS infection is progressive, even in a neurological asymptomatic stage. (22) However, CSF pleocytosis decreased in PLWH with severe immunosuppression, (15, 19, 21) probably because of T-cell deficiency.

Monotherapy ART with zidovudine, the only existing drug at the beginning of the AIDS epidemic, resulted in a 53 to 57% decrease in CSF neopterin concentrations. (23) The next drug on the market, didanosine, had no such effect. (24). Zidovudine-resistant variants in the brain developed during monotherapy. (25) It was obvious that combination treatment was necessary to avoid viral resistance, and in 1996, when protease inhibitors was added to 2 nucleoside reverse transcriptase inhibitors (NRTI), a breakthrough in HIV care was achieved. Blood HIV load decreased to numbers below 50 copies/ml in most PLWH, and those with HIV-related symptoms clinically improved.

For peer review only

We found that ART was very effective in reducing viral load in CSF. Ultrasensitive PCR showed that highly effective combination treatment (HAART) resulted in undetectable HIV-1 RNA copies in CSF, although the virus was still detectable in plasma. (12) (26). Several ART combinations proved virologically effective in CSF, (26-29) and reduced blood-brain barrier integrity and intrathecal immunoglobulin production. (30, 31). CSF markers of axonal injury were also normalized. (13, 17, 28, 32) .

Studies of the highly effective short-term effects of ART were followed by long-term studies. We found that CSF viral loads were effectively suppressed over long periods of observation, but CSF signs of slight immune activation were still present in many PLWH after several years of suppressive treatment. (14, 16, 33)

While CNS infection is generally well controlled by systemic suppressive ART, there are exceptions when the HIV RNA load increases in the CSF despite suppression of the plasma viral load, a phenomenon referred to as asymptomatic CSF viral escape. (34) In these PLWH, CSF viral counts often reach just above 50 copies/ml without accompanying CSF pleocytosis or CSF signs of neuronal injury, measured as increased CSF concentration of Nfl. The lack of clinical symptoms, and the fact that the viral CSF increase was most often transient and reversed without changing therapy, has resulted in this condition to be interpreted as benign “CSF viral blips”, similar to plasma blips. (35) CSF escape is associated with increased CSF neopterin concentrations and may be related to the size of the CNS HIV reservoir. Correspondingly, residual CSF viral loads below the limit of quantification by standard assays also correlate with the degree of CSF immune activation in PLWH receiving suppressive ART. (26, 36) This reinforces the view that intrathecal immune activation is driven by persistent virus in the CNS. Nevertheless, similar to findings during

For peer review only

systemic infection, (37) treatment intensification does not seem to decrease the residual CSF viral load or inflammation, (38, 39) suggesting that there is no ongoing HIV replication during effective treatment.

Although it has not yet been definitely proven, data suggest that a stable permanent infection of cells in the CNS is established later than in the systemic viral reservoirs, (40, 41) which are highly concentrated in memory T-cell compartments within the first days of systemic HIV infection. (42) When examining anti-HIV antibody formation as a surrogate marker for antigen load and the size of the viral reservoir (43) in patients followed longitudinally during early HIV infection, serum anti-HIV antibodies emerged in blood by day 30 in untreated early infection, while CSF antibodies reached similar levels about 2 weeks later. (44) In addition, high antibody levels, comparable to those observed in chronically infected subjects, were reached several months later in CSF, as compared with blood. In addition, while treatment of chronic infection resulted in only small reductions in levels of anti-HIV antibodies in both CSF and serum, treatment during early infection substantially reduced CSF levels of antibodies, sometimes to levels close to those in HIV-negative controls. In contrast, antibody levels in serum were less affected, (44) altogether supporting this hypothesis. Our findings and those from other groups further support compartmentalization of HIV infection and immune activation in the CNS. (45-48)

The low-grade CNS immune activation found during suppressive ART may not be solely ascribed to HIV itself, since co-morbidities, co-infections, and life-style related factors can contribute, as elegantly shown in the COBRA study. (49, 50) The importance of appropriate controls was also demonstrated in our HIV-negative PrEP controls in whom immune

activation markers and signs of neuronal injury increased as compared to non-PrEP controls. (51)

Occasionally, CSF viral escape, i.e, viral load in CSF but not in blood, is accompanied by HIV-induced neurological and neurocognitive signs and symptoms, which have been defined as "symptomatic CSF escape". (52-54) Another phenomenon that causes increased CSF viral load in well-treated PLWH is a concomitant infection in the nervous system. As an example, herpes zoster sometimes results in an inflammatory CSF reaction with slight pleocytosis, increased CSF neopterin concentrations, and increased CSF viral load, but with no detectable plasma virus. This phenomenon has been called "secondary CSF escape" and may be the result of latent virus released or detected from activated monocytes. (55)

DISCUSSION

HIV-1 infects many compartments in the human body, including the CNS. CSF surrounds the brain and is a fluid accessible to LP, which can give valuable information on infectious activity and pathological processes in the CNS. As noted earlier, in our clinical cohort most participants were asymptomatic. It can be a challenge to enroll PLWH to do repeated LP. For this reason, most cohorts studying CSF only include participants with neurological and cognitive complications, or opportunistic CNS infections having limited follow-up. To survey the whole panorama of the infectious course, we have a long-lasting collaboration with other centers, the most important being cohorts at UCSF, San Francisco, CA, USA, and Milan Italy. This enables us to compare our cohort with a large number of PLWH suffering from AIDS dementia complex and other CNS complications.

For peer review only

HIV infection changed dramatically in severity since we began this study in 1985. At that time, we had no idea that a combination of antiretroviral drugs could have such impact. The natural course of the infection was observed with considerable data showing progressive CNS disease in several PLWH. When ART was introduced, the longitudinal project changed to monitor whether CSF viral load, markers of inflammation, and CNS injury became normalized. Some early medication with monotherapy had a limited short-term effect. It was not until 1996 when highly active antiretroviral treatment (HAART) with 3 drugs begun that the disease became a chronic latent infection with a long-life expectancy and a high quality of life. However, PLWH must remain life-long on ART. Luckily, up to now there are many modern drugs to choose from with no or minor adverse events.

There has been a debate over whether the most frequently used criteria for cognitive impairment in people with HIV, namely the Frascati criteria developed in 2007 (56) overestimate cognitive impairment. New criteria that are more appropriate for the modern era have been sought. (57, 58) The significance of mild forms of neurocognitive disorders and asymptomatic cognitive impairment detected in well-treated PLWH are controversial. Furthermore, if this condition exist, are they progressive or reversible?(2, 59). Increased CSF inflammation has been reported in PLWH on suppressive ART who experience mild cognitive impairment, (60) but the implications of this are yet to be settled. The role of aging, underlying diseases, and life-style factors of mild neurocognitive disorder are largely unknown. In such patients, longitudinal CSF studies are helpful to determine pathogenic factors that may affect the CNS.

Despite our intention to follow participants annually, it was difficult to accomplish. Some participants consented to repeated LP, but at longer intervals than annually. Moreover, the

For peer review only - http://bmjopen.bmj.com/site/about/guidelines.xhtml

Covid-19 pandemic halted several clinical studies for two years. Another limitation is that our cohort included relatively few PLWH with advanced disease and CNS complications. By means of international collaborations, more CSF data from patients with severe neurological complications and opportunistic CNS infections have been used for comparison with our cohort data in several cross-sectional studies. A limitation is also the infrequent use of extensive neuropsychiatric test batteries. Several attempts to include regular neuropsychiatric tests in the protocol failed due to methodological difficulties and a lack of resources. Furthermore, it was difficult to enroll a valid control group. Another complication while managing neuropsychiatric analyses in longitudinal studies involving repeated tests is the learning factor, which may result in false test results. In addition, there were many participants with language difficulties.

A major strength of our study is its uniquely long follow-up time, with CSF data from a population of PLWH with a predominately neuroasymptomatic clinical appearance, which we believe has never been done before. In addition, the study was performed with a very strict protocol for collecting and storing CSF/blood samples at one center, and only engaging a handful of clinicians to enhance consistency and uniformity.

Our future objectives involve several questions. Are there any active, ongoing inflammatory or neurotoxic processes remaining in the brain, despite successful virological treatment? Are there any complications from the CNS caused by chronic antiretroviral medication? Characterization of the CNS HIV reservoir and its establishment is still largely unknown. What is the importance of compartmentalized CNS infection, and if peripheral eradication treatment in the blood and the lymphatic system proves successful in the future, is it possible

For peer review only

bring about the same effect in the CNS? The study will continue with follow-up of already included participants and recruitment of newly diagnosed PLWH.

Ethics

At the time of HIV diagnosis, each person being diagnosed is asked for consent to be included in the study, and to decline without stating any reasons. Participants always have the right to exit the study. They may also request an extract of their data from the register, at no charge, in accordance with the European General Data Protection Regulation (GDPR 2016/679) and the Swedish Data Protection Act (2018:218). The study is approved by the Regional Ethics Review Board in Gothenburg, Sweden (Ö588-01) and performed in accordance with the Helsinki Declaration. All participants gave their informed consent.

Biobank: Sahlgrenska University Hospital, Gothenburg, Sweden, no. 890

International collaborations: Collaborations with HIV cohorts from other countries: University of California (UCSF); Prof. Richard W Price; University of Milano, Italy; Prof. Paola Cinque; University of Sydney, Australia; Prof. Bruce Brew; University of Innsbruck, Austria, Prof. Dietmar Fuchs; University of North Carolina (UNC), Chapel Hill; Prof. Ron Swanstrom.

Author affiliation: Department of Infectious Diseases, Institute of Biomedicine, Sahlgrenska Academy, University of Gothenburg, Gothenburg, Sweden; and Region Västra Götaland, Sahlgrenska University Hospital, Gothenburg, Sweden.

For peer review only

Authors contribution: LH and MG designed the study, examined and collected CSF from the majority of the participants, registered the results, and wrote the paper with equal contribution.

Funding to report for this paper: This work was supported by the Swedish state under an agreement between the Swedish government and the county councils (grant ALFGBG-965885).

Competing interests: LH has no competing interest. MG has received research grants from Gilead Sciences and Janssen-Cilag, and honoraria as a speaker and/or scientific advisor from Amgen, AstraZeneca, Biogen, Bristol-Myers Squibb, Gilead Sciences, GlaxoSmithKline/ViiV, Janssen-Cilag, MSD, Novocure, Novo Nordic, Pfizer and Sanofi.

Patient consent for publication: Not required

Provenance and peer review: Not commissioned; externally peer reviewed.

Data availability statement: Data can be made available upon legitimate request pending ethical approval.

For peer review only

References

1. Dore GJ, Correll PK, Li Y, et al. Changes to AIDS dementia complex in the era of highly active antiretroviral therapy. *AIDS* 1999;13(10):1249–53.

2. McArthur JC, Brew BJ, Nath A. Neurological complications of HIV infection. *Lancet Neurol* 2005;4(9):543–55.

3. Hagberg L, Malmvall BE, Svennerholm L, et al. Guillain-Barre syndrome as an early manifestation of HIV central nervous system infection. *Scand J Infect Dis* 1986;18(6):591–2.

4. Chiodi F, Asjo B, Fenyo EM, et al. Isolation of human immunodeficiency virus from cerebrospinal fluid of antibody-positive virus carrier without neurological symptoms. *Lancet* 1986;2(8518):1276–7.

5. Dunlop O, Bjorklund RA, Abdelnoor M, et al. Five different tests of reaction time evaluated in HIV seropositive men. *Acta Neurol Scand* 1992;86(3):260–6.

6. Mellgren A, Norkrans G, Hagberg L, et al. Slowed reaction time in HIV-1-infected patients without AIDS. *Acta Neurol Scand* 2000;102(3):169–74.

7. Cysique LA, Maruff P, Darby D, et al. The assessment of cognitive function in advanced HIV-1 infection and AIDS dementia complex using a new computerised cognitive test battery. A*rch Clin Neuropsychol* 2006;21(2):185–94.

8. Maruff P, Thomas E, Cysique L, et al. Validity of the CogState brief battery: relationship to standardized tests and sensitivity to cognitive impairment in mild traumatic brain injury, schizophrenia, and AIDS dementia complex. *Arch Clin Neuropsychol* 2009;24(2):165–78.

9. Yilmaz A, Mellgren A, Fuchs D, et al. Switching from a regimen containing abacavir/lamivudine or emtricitabine/tenofovir disoproxil fumarate to emtricitabine/tenofovir alafenamide fumarate does not affect central nervous system HIV-1 infection. *Infect Dis (Lond)* 2019;51(11–12):838–46.

For peer review only

10. Chiodi F, Keys B, Albert J, et al. Human immunodeficiency virus type 1 is present in the cerebrospinal fluid of a majority of infected individuals. *J Clin Microbiol* 1992;30(7):1768–71.

11. Gisslen M, Hagberg L, Fuchs D, et al. Cerebrospinal fluid viral load in HIV-1-infected patients without antiretroviral treatment: a longitudinal study. *J Acquir Immune Defic Syndr Hum Retrovirol* 1998;17(4):291–5.

12. Gisslen M, Norkrans G, Svennerholm B, et al. HIV-1 RNA detectable with ultrasensitive quantitative polymerase chain reaction in plasma but not in cerebrospinal fluid during combination treatment with zidovudine, lamivudine and indinavir. *AIDS* 1998;12(1):114–6.

13. Abdulle S, Mellgren A, Brew BJ, et al. CSF neurofilament protein (NFL)—a marker of active HIV-related neurodegeneration. *J Neurol* 2007;254(8):1026–32.

14. Eden A, Price RW, Spudich S, et al. Immune activation of the central nervous system is still present after > 4 years of effective highly active antiretroviral therapy. *J Infect Dis* 2007;196(12):1779–83.

15. Hagberg L, Forsman A, Norkrans G, et al. Cytological and immunoglobulin findings in cerebrospinal fluid of symptomatic and asymptomatic human immunodeficiency virus (HIV) seropositive patients. *Infection* 1988;16(1):13–8.

16. Ulfhammer G, Eden A, Mellgren A, et al. Persistent central nervous system immune activation following more than 10 years of effective HIV antiretroviral treatment. *AIDS* 2018;32(15):2171–8.

17. Jessen Krut J, Mellberg T, Price RW, et al. Biomarker evidence of axonal injury in neuroasymptomatic HIV-1 patients. *PLoS One* 2014;9(2):e88591.

18. Andersson LM, Svennerholm B, Hagberg L, et al. Higher HIV-1 RNA cutoff level required in cerebrospinal fluid than in blood to predict positive HIV-1 isolation. *J Med Virol* 2000;62(1):9–13.

For peer review only

19. Gisslen M, Fuchs D, Svennerholm B, et al. Cerebrospinal fluid viral load, intrathecal immunoactivation, and cerebrospinal fluid monocytic cell count in HIV-1 infection. *J Acquir Immune Defic Syndr* 1999;21(4):271–6.

20. Spudich SS, Nilsson AC, Lollo ND, et al. Cerebrospinal fluid HIV infection and pleocytosis: relation to systemic infection and antiretroviral treatment. *BMC Infect Dis* 2005;5:98.

21. Ulfhammer G, Eden A, Antinori A, et al. Cerebrospinal fluid viral load across the spectrum of untreated HIV-1 infection: a cross-sectional multi-center study. *Clin Infect Dis* 2022;75(3):493–502.

22. Gisslen M, Chiodi F, Fuchs D, et al. Markers of immune stimulation in the cerebrospinal fluid during HIV infection: a longitudinal study. *Scand J Infect Dis* 1994;26(5):523–33.

23. Hagberg L, Norkrans G, Andersson M, et al. Cerebrospinal fluid neopterin and beta 2-microglobulin levels in neurologically asymptomatic HIV-infected patients before and after initiation of zidovudine treatment. *Infection* 1992;20(6):313–5.

24. Hagberg L, Norkrans G, Gisslen M, et al. Intrathecal immunoactivation in patients with HIV-1 infection is reduced by zidovudine but not by didanosine. *Scand J Infect Dis* 1996;28(4):329–33.

25. Di Stefano M, Norkrans G, Chiodi F, et al. Zidovudine-resistant variants of HIV-1 in brain. *Lancet* 1993;342(8875):865.

26. Yilmaz A, Svennerholm B, Hagberg L, et al. Cerebrospinal fluid viral loads reach less than 2 copies/ml in HIV-1-infected patients with effective antiretroviral therapy. *Antivir Ther* 2006;11(7):833–7.

27. Gisslen M, Svennerholm B, Fuchs D, et al. Neurological efficacy of stavudine, zidovudine, and lamivudine. *Lancet* 1998;352(9125):402–3.

For peer review only

28. Mellgren A, Price RW, Hagberg L, et al. Antiretroviral treatment reduces increased CSF neurofilament protein (NFL) in HIV-1 infection. *Neurology* 2007;69(15):1536–41.

29. Yilmaz A, Stahle L, Hagberg L, et al. Cerebrospinal fluid and plasma HIV-1 RNA levels and lopinavir concentrations following lopinavir/ritonavir regimen. *Scand J Infect Dis* 2004;36(11–12):823–8.

30. Abdulle S, Hagberg L, Gisslen M. Effects of antiretroviral treatment on blood-brain barrier integrity and intrathecal immunoglobulin production in neuroasymptomatic HIV-1-infected patients. *HIV Med* 2005;6(3):164–9.

31. Anesten B, Zetterberg H, Nilsson S, et al. Effect of antiretroviral treatment on blood-brain barrier integrity in HIV-1 infection. *BMC Neurol* 2021;21(1):494.

32. Yilmaz A, Blennow K, Hagberg L, et al. Neurofilament light chain protein as a marker of neuronal injury: review of its use in HIV-1 infection and reference values for HIV-negative controls. *Expert Rev Mol Diagn* 2017;17(8):761–70.

33. Yilmaz A, Yiannoutsos CT, Fuchs D, et al. Cerebrospinal fluid neopterin decay characteristics after initiation of antiretroviral therapy. *J Neuroinflammation* 2013;10:62.

34. Eden A, Fuchs D, Hagberg L, et al. HIV-1 viral escape in cerebrospinal fluid of subjects on suppressive antiretroviral treatment. *J Infect Dis* 2010;202(12):1819–25.

35. Eden A, Nilsson S, Hagberg L, et al. Asymptomatic cerebrospinal fluid HIV-1 viral blips and viral escape during antiretroviral therapy: a longitudinal study. *J Infect Dis* 2016;214(12):1822–5.

36. Dahl V, Peterson J, Fuchs D, et al. Low levels of HIV-1 RNA detected in the cerebrospinal fluid after up to 10 years of suppressive therapy are associated with local immune activation. *AIDS* 2014;28(15):2251–8.

For peer review only

37. Dinoso JB, Kim SY, Wiegand AM, et al. Treatment intensification does not reduce residual HIV-1 viremia in patients on highly active antiretroviral therapy. *Proc Natl Acad Sci USA* 2009;106(23):9403–8.

38. Dahl V, Lee E, Peterson J, et al. Raltegravir treatment intensification does not alter cerebrospinal fluid HIV-1 infection or immunoactivation in subjects on suppressive therapy. *J Infect Dis* 2011;204(12):1936–45.

39. Yilmaz A, Verhofstede C, D'Avolio A, et al. Treatment intensification has no effect on the HIV-1 central nervous system infection in patients on suppressive antiretroviral therapy. *J Acquir Immune Defic Syndr* 2010;55(5):590–6.

40. Hellmuth J, Slike BM, Sacdalan C, et al. Very early initiation of antiretroviral therapy during acute HIV infection is associated with normalized levels of immune activation markers in cerebrospinal fluid but not in plasma. *J Infect Dis* 2019;220(12):1885–91.

41. Gisslen M, Hunt PW. Antiretroviral treatment of acute HIV infection normalizes levels of cerebrospinal fluid markers of central nervous system (CNS) inflammation: a consequence of a reduced CNS reservoir? *J Infect Dis* 2019;220(12):1867–9.

42. Hunt PW, Lee SA, Siedner MJ. Immunologic biomarkers, morbidity, and mortality in treated HIV infection. *J Infect Dis* 2016;214 Suppl 2:S44–50.

43. Lee SA, Bacchetti P, Chomont N, et al. Anti-HIV antibody responses and the HIV reservoir size during antiretroviral therapy. *PLoS One* 2016;11(8):e0160192.

44. Burbelo PD, Price RW, Hagberg L, et al. Anti-human immunodeficiency virus antibodies in the cerebrospinal fluid: evidence of early treatment impact on central nervous system reservoir? *J Infect Dis* 2018;217(7):1024–32.

45. Bednar MM, Sturdevant CB, Tompkins LA, et al. Compartmentalization, viral evolution, and viral latency of HIV in the CNS. *Curr HIV/AIDS Rep* 2015;12(2):262–71.

For peer review only

46. Joseph SB, Kincer LP, Bowman NM, et al. Human immunodeficiency virus type 1 RNA detected in the central nervous system (CNS) after years of suppressive antiretroviral therapy can originate from a replicating CNS reservoir or clonally expanded cells. *Clin Infect Dis* 2019;69(8):1345–52.

47. Gisslen M, Svennerholm B, Norkrans G, et al. Cerebrospinal fluid and plasma viral load in HIV-1-infected patients with various anti-retroviral treatment regimens. *Scand J Infect Dis* 2000;32(4):365–9.

48. Gisslen M, Keating SM, Spudich S, et al. Compartmentalization of cerebrospinal fluid inflammation across the spectrum of untreated HIV-1 infection, central nervous system injury and viral suppression. *PLoS One* 2021;16(5):e0250987.

49. De Francesco D, Wit FW, Burkle A, et al. Do people living with HIV experience greater age advancement than their HIV-negative counterparts? *AIDS* 2019;33(2):259–68.

50. Booiman T, Wit FW, Maurer I, et al. High cellular monocyte activation in people living with human immunodeficiency virus on combination antiretroviral therapy and lifestyle-matched controls is associated with greater inflammation in cerebrospinal fluid. *Open Forum Infect Dis* 2017;4(3):ofx108.

51. Robertson J, Eden A, Nystrom K, et al. Increased immune activation and signs of neuronal injury in HIV-negative people on preexposure prophylaxis. *AIDS* 2021;35(13):2129–36.

52. Canestri A, Lescure FX, Jaureguiberry S, et al. Discordance between cerebral spinal fluid and plasma HIV replication in patients with neurological symptoms who are receiving suppressive antiretroviral therapy. *Clin Infect Dis* 2010;50(5):773–8.

53. Peluso MJ, Ferretti F, Peterson J, et al. Cerebrospinal fluid HIV escape associated with progressive neurologic dysfunction in patients on antiretroviral therapy with well controlled plasma viral load. *AIDS* 2012;26(14):1765–74.

For peer review only

54. Ferretti F, Gisslen M, Cinque P, et al. Cerebrospinal fluid HIV escape from antiretroviral therapy. *Curr HIV/AIDS Rep* 2015;12(2):280–8.

55. Hagberg L, Price RW, Zetterberg H, et al. Herpes zoster in HIV-1 infection: the role of CSF pleocytosis in secondary CSF escape and discordance. *PLoS One.* 2020;15(7):e0236162.

56. Antinori A, Arendt G, Becker JT, et al. Updated research nosology for HIV-associated neurocognitive disorders. *Neurology* 2007;69(18):1789–99.

57. Gisslen M, Price RW, Nilsson S. The definition of HIV-associated neurocognitive disorders: are we overestimating the real prevalence? *BMC Infect Dis* 2011;11:356.

58. Nightingale S, Dreyer AJ, Saylor D, et al. Moving on from HAND: why we need new criteria for cognitive impairment in persons living with human immunodeficiency virus and a proposed way forward. *Clin Infect Dis* 2021;73(6):1113–8.

59. Robertson KR, Smurzynski M, Parsons TD, et al. The prevalence and incidence of neurocognitive impairment in the HAART era. *AIDS* 2007;21(14):1915–21.

60. Eden A, Marcotte TD, Heaton RK, et al. Increased intrathecal immune activation in virally suppressed HIV-1 infected patients with neurocognitive impairment. *PLoS One* 2016;11(6):e0157160.

For peer review only

Figure 1. Flow chart of 662 people living with HIV (PLWH) included in the Gothenburg HIV CSF study (1985 to Apr 7, 2022).

For peer review only

For peer review only

Figure 1

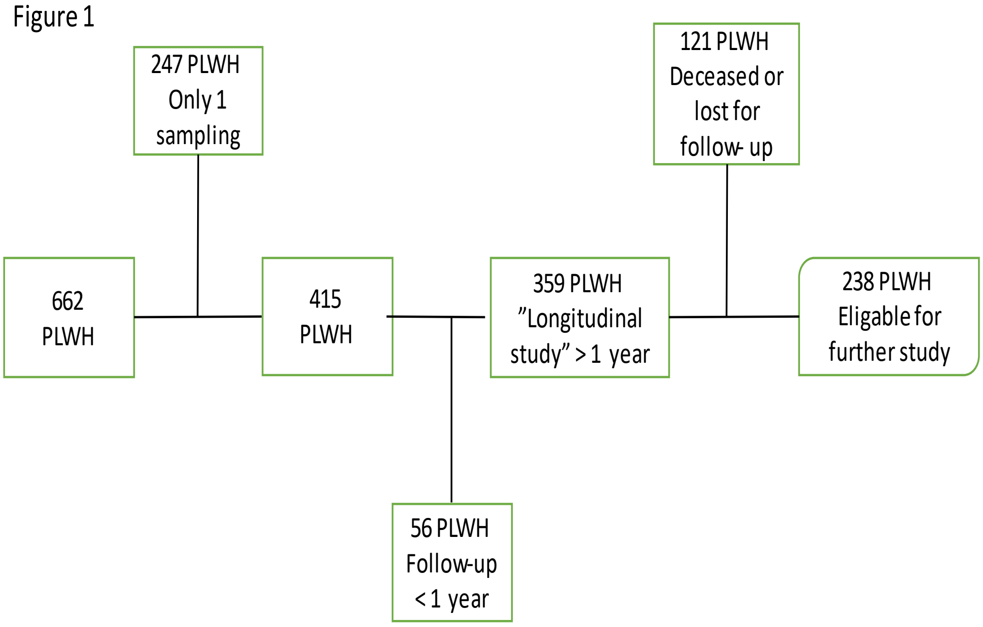
247 PLWH
Only 1
sampling
121 PLWH
Deceased or
lost for
follow- up
662
PLWH
415
PLWH
359 PLWH
"Longitudinal
study" > 1 year
238 PLWH
Eligable for
further study
56 PLWH
Follow-up
< 1 year

BMJ Open

Cohort profile: A longitudinal study of HIV infection in the central nervous system with focus on cerebrospinal fluid- The Gothenburg HIV CSF Study Cohort

Journal:	*BMJ Open*
Manuscript ID	bmjopen-2022-070693.R1
Article Type:	Cohort profile
Date Submitted by the Author:	31-Mar-2023
Complete List of Authors:	Hagberg, Lars; University of Gothenburg, Department of Infectious Diseases<br>Gisslén, Magnus; University of Gothenburg Sahlgrenska Academy, Department of Infectious Diseases, Institute of Biomedicine; Sahlgrenska University Hospital, Department of Infectious Diseases, Region Västra Götaland
**Primary Subject Heading**:	Infectious diseases
Secondary Subject Heading:	HIV/AIDS
Keywords:	HIV & AIDS < INFECTIOUS DISEASES, MICROBIOLOGY, Infectious disease/HIV < NEUROLOGY

SCHOLARONE™
Manuscripts

I, the Submitting Author has the right to grant and does grant on behalf of all authors of the Work (as defined in the below author licence), an exclusive licence and/or a non-exclusive licence for contributions from authors who are: i) UK Crown employees; ii) where BMJ has agreed a CC-BY licence shall apply, and/or iii) in accordance with the terms applicable for US Federal Government officers or employees acting as part of their official duties; on a worldwide, perpetual, irrevocable, royalty-free basis to BMJ Publishing Group Ltd ("BMJ") its licensees and where the relevant Journal is co-owned by BMJ to the co-owners of the Journal, to publish the Work in this journal and any other BMJ products and to exploit all rights, as set out in our licence.

The Submitting Author accepts and understands that any supply made under these terms is made by BMJ to the Submitting Author unless you are acting as an employee on behalf of your employer or a postgraduate student of an affiliated institution which is paying any applicable article publishing charge ("APC") for Open Access articles. Where the Submitting Author wishes to make the Work available on an Open Access basis (and intends to pay the relevant APC), the terms of reuse of such Open Access shall be governed by a Creative Commons licence – details of these licences and which Creative Commons licence will apply to this Work are set out in our licence referred to above.

Other than as permitted in any relevant BMJ Author's Self Archiving Policies, I confirm this Work has not been accepted for publication elsewhere, is not being considered for publication elsewhere and does not duplicate material already published. I confirm all authors consent to publication of this Work and authorise the granting of this licence.

For peer review only

BMJ Open Cohort profile

Title: Cohort profile: A longitudinal study of HIV infection in the central nervous system with focus on cerebrospinal fluid-The Gothenburg HIV CSF Study Cohort

Authors: Lars Hagberg[1,2], Magnus Gisslén[1,2]

1. Department of Infectious Diseases, Institute of Biomedicine, Sahlgrenska Academy, University of Gothenburg, Gothenburg, Sweden
2. Region Västra Götaland, Sahlgrenska University Hospital, Gothenburg, Sweden

Corresponding author: Lars Hagberg.

Postal address: Dept of Infectious Diseases, Sahlgrenska University Hospital SE 416 85 Gothenburg, Sweden.

Email: lars.hagberg@gu.se

Word count: 3373

For peer review only

ABSTRACT

Purpose: In order to enable long-term follow-up of the natural course of HIV infection in the central nervous system, a longitudinal cohort study with repeated cerebrospinal fluid (CSF) analyses at intervals over time was initiated in 1985. When antiretrovirals against HIV were introduced in the late 1980s, short- and long-term effects of various antiretroviral treatment (ART) regimens were added to the study.

Participants: All adults people living with HIV (PLWH) who were diagnosed at or referred to the Department of Infectious Diseases, Sahlgrenska University Hospital, Gothenburg, Sweden were asked to participate in the Gothenburg HIV CSF Study Cohort. PLWH with neurological symptoms or other clinical symptoms of HIV, as well as those with no symptoms of HIV infection were included. Most participants were asymptomatic which distinguishes this cohort from most other international HIV CSF studies. In addition, HIV-negative controls were recruited. These included people on HIV pre-exposure prophylaxis who served as life-style matched controls to HIV-infected men who have sex with men. Since lumbar puncture (LP) is an invasive procedure, some PLHW only consented to participate in one examination. Furthermore, at the beginning of the study several participants were lost to follow-up having died from AIDS. Of 662 PLWH where an initial LP was done, 415 agreed to continue with follow-up. Among the 415, 56 only gave permission to be followed with LP for less than 1 year, mainly to analyze the short-term effect of ART. The remaining 359 PLWH were followed-up with repeated LP for periods ranging from > 1 to 30 years. This group was defined as the "longitudinal cohort". So far, on 7 April 2022, 2,650 LP and samplings of paired CSF/blood had been performed, providing a unique biobank.

Findings to date: A general finding during the 37-year study period was that HIV infection in the central nervous system, as mirrored by CSF findings, appears early in the infectious

For peer review only

course of the disease, and progresses slowly in the vast majority of untreated PLWH. Combination ART has been highly effective in reducing CSF viral counts, inflammation, and markers of neural damage. Minor CSF signs of long-term sequels or residual inflammatory activity and CSF escape (viral CSF blips) have been observed during follow-up. The future course of these changes and their clinical impact require further studies.

Future plans: PLWH today have a life expectancy close to that of non-infected people**.** Therefore, our cohort provides a unique opportunity to study the long-term effects on HIV infection in the central nervous system and the impact of ART, and is an ongoing study.

Keywords: HIV infection, central nervous system, cerebrospinal fluid

Strengths and limitations

- Strength of our study is its uniquely long follow-up time, with CSF data from a population of PLWH with a predominately neuroasymptomatic clinical appearance.
- A strict protocol for collecting and storing CSF/blood samples at one center, and only engaging a handful of clinicians to enhance consistency and uniformity is another strength.
- Difficult to follow the protocol with yearly repeated LP in several participants due to consent and Covid-19 pandemic is a limitation.
- Infrequent use of extensive neuropsychiatric testing.
- Only limited number of participants suffering from severe neurological complications and opportunistic CNS infections

For peer review only

INTRODUCTION

Chronic untreated HIV infection causes progressive immunodeficiency that leads to AIDS in a median of 10 or 11 years after a primary infection. Before effective treatment was available, HIV-associated dementia was frequently observed in the late stages of the infection. The introduction of combination antiretroviral treatment (ART), that preserves or restores immune functions has had a major impact on morbidity and mortality. It has also resulted in a marked reduction of HIV-associated dementia and other neurologic complications. (1) Nevertheless, mild forms of HIV-associated neurocognitive disorders have frequently been reported even during suppressive ART. (2) The research questions for the present study have been: Can biomarkers in cerebrospinal fluid (CSF) be used to chart the natural course of central nervous system (CNS) HIV infection, and can we determine the short and long-term effects of ART?

Cohort description and method

Study population

The study was initiated in 1985 when two patients attending our clinic presented with Guillain Barre syndrome as a complication to HIV infection and exhibited HIV isolated in the CSF. (3) Later we found that HIV-1 could also be isolated in CSF of virus carriers who had no neurological symptoms. (4) At that time, it was not known that HIV was neurotropic.

Since 1985, HIV-infected people living with HIV (PLWH) in the Gothenburg area of Sweden have been enrolled in a longitudinal study with serial sampling of CSF and blood. Lumbar punctures (LP) are performed annually, if possible, or more frequently in connection with introduction or cessation of ART. Both PLWH with neurological or other clinical symptoms of HIV, and asymptomatic PLWH have been included in the study.

For peer review only

Of 662 PLWH in whom an initial LP was done, 415 PLWH agreed to continue with follow-up. Among the 415, 56 only gave permission to be followed with LP for less than 1 year, mainly to analyze the short-term effect of ART. The remaining 359 PLWH were followed with repeated LP for periods > 1 up to 30 years. This group was defined as the "longitudinal cohort" (see flow chart, Fig.1). The 247 PLWH who participated with only one CSF/blood sampling have been included in many cross-sectional studies. In this ongoing cohort study we present data from 1985 to 7 April 2022 including 2,650 paired CSF/blood samples, providing a unique and continuously growing biobank.

Longitudinal cohort

In total, 359 PLWH were followed with a mean number of 6.26 (range 2–30) LP and CSF/blood analyses over a mean period of 6.89 years (range 1–30 years). Patient characteristics, time of follow-up, and number of CSF analyses is shown in Table 1, and time for inclusion and number PLWH still eligible for follow up is shown in table 2.

Patient and Public Involvement

Development of the research question, outcome measures, and presentation of results has been done with the local PLWH organization PG Vast.

Table 1. Longitudinal cohort characterization

Number of participants followed > 1 year:	359
Mean time of follow-up:	6.89 years (range 1–30)
Number of lumbar punctures/individuals:	6.26 (range 2–30)
Age at inclusion, mean:	40.7 years (range 17–73)
Gender, male/female:	247/112

For peer review only

Geographic background (n):	Sweden 182
	Europe (outside Sweden) 37
	Africa 93
	Asia 35
	Middle East 3
	America 9

HIV1/HIV2	356/3
mean $CD4^+$ cells at inclusion:	369 $x10^6/L$ (range 0–2131)
CDC classification[1] at inclusion:	A1-A3 n = 244; B1-B3 n = 34; C1-C3 (AIDS) n = 81

ART at inclusion (n):	No treatment: 298 (of whom 31 had primary infection)
	Treatment suppressed: 49
	Treatment failure: 11
	Treatment interruption: 1

Neurology at inclusion (n):	Neuroasymptomatic: 328
	CNS opportunist: 12
	HIV-associated dementia: 10
	Other CNS complications: 9

[1]Revised classification system for HIV: MMWR Dec 18; 41:1-19. 1993

For peer review only

Table 2. Time for inclusion (5 years interval) in the cohort study, number of LP:s, and number of PLWH still eligible for follow up.

PLWH No	LP No (range)		PLWH still eligible No
1985-1990	36	247 (2-20)	5
1990-1995	32	216 (2-30)	7
1995-2000	54	442 (2-27)	22
2000-2005	48	396 (2-19)	33
2005-2010	48	328 (2-19)	40
2010-2015	62	326 (2-16)	57
2015-2020	74	292 (2-7)	67

About half of the participants (51%) were born in Sweden (n = 182) and 177 (49%) outside Sweden, most commonly in sub-Saharan Africa (n = 93) (26%), Europe outside Sweden (n = 37) (10%), and Asia (n = 35) (10%). The Covid-19 pandemic partially halted the study for a period. However, 166 PLWH (46%) had been followed for more than 5 years and 65 of these (18%) have been followed for more than 10 years, providing great opportunities for longitudinal evaluations. A total of 121 PLWH were diseased or lost to follow-up. The remaining 238 PLWH are eligible to ask for further follow-up (see flow chart Fig. 1).

In addition, 94 HIV-negative healthy controls have been recruited for CSF/blood sampling, of whom 53 are men on HIV pre-exposure prophylaxis (PrEP). Follow-up sampling of this cohort is ongoing.

Validation of the study population

For peer review only

PLWH whose CSF/blood was sampled only once and declined to continue the study because of discomfort from the initial LP, or were too sick for follow-up, or were below 18 years of age, or were lost to follow-up were registered in the reject log. That log included 247 PLWH with a mean age of 40.1 years (range 2–76), of whom 74 (30%) were women and 173 (70%) were male. Their mean CD4 cell count was 344 x10^6/L, (range 10–1420); 40% were classified as CDC stage C1-C3 (AIDS). The number of AIDS patients (40%) were larger than the longitudinal cohort group (22%), but otherwise age, gender, and CD4 cell count was of similar magnitude between the groups.

Cohort variables and laboratory analyses

Full clinical history, including co-morbidity, treatment, and laboratory results were collected throughout the study. Variables recorded at enrolment included sex, country of birth, mode of HIV transmission, date of last HIV-negative test (if any), and first positive HIV-test, and suspected country for HIV transmission. Data collected/updated at each follow-up visit included ART, prophylaxis of opportunistic infections, and co-medications administered. Data on co-infection with hepatitis C and B virus, weight, date and type of AIDS-defining events and non-AIDS events, and date and cause of death were also included. HIV-RNA, CD4+ and CD8+ T-cell counts and CD4/CD8 ratios, HIV subtype and HIV drug resistance (including viral sequences) were also recorded.

CSF and blood collection took place in the morning, before breakfast in a standardized manner with the subject in a lateral recumbent position. Twenty-four mL of CSF were collected and centrifuged for cell counting. CSF cells and buffy coat were stored separately, and cell-free CSF, serum, and plasma were divided into fractions. Fractions not immediately analyzed were stored at –70°C in the local laboratory after collection. In conjunction with

For peer review only

each LP. virological, immunological, and neuronal injury markers in the CSF were compared with the clinical course. The laboratory analyses were grouped in three categories.

1. Virology
2. Markers of inflammation and immunology
3. Markers of CNS injury

1. *Virology*: Since its introduction in 1996 quantitative HIV-1 RNA polymerase chain reaction (PCR) has been used as the main marker of viral load (currently Cobas Taqman v.2, Roche Diagnostic Systems, Hoffmann-La Roche, Inc., Basel, Switzerland). Quantitative HIV-1 DNA real-time PCR (TaqMan5´nuclease) assay has been analyzed in sub-populations. Prior to 1996, CSF HIV antigen test and virus isolation were included in the protocol.

2. *Markers of inflammation and immunology*; Neopterin concentration reflects macrophage activity and has been the main marker of CSF inflammation (RIA method Henning test Neopterin, BRAHMS, Berlin Germany). Among other procedures CSF monocyte cell count, protein electrophoresis, oligoclonal bands, and beta-2-microglobulin concentration have been analyzed in all patients. Various cytokines such as MCP-1, IP-10, CXCL10, uPA, and suPAR have also been measured in sub-populations.

3. *Markers of CNS injury*: CSF neurofilament light protein (Nfl) concentration that reflects axonal damage has been the main marker used to estimate CNS injury in CSF (NF-light ELISA kit; Uman Diagnostics AB, Umeå, Sweden), and ultrasensitive single molecule array (Simoa) method has been used for blood. Other markers such as

For peer review only

gangliosides (GM1, GD1a, GD1b, and GT1b), sulfatides, glial markers, including GFAP (astroglia) and GD3 (microglial/macrophages), t-tau, p-tau, beta-amyloids, s-APP and neurogranin (synapse protein) have been studied in sub-populations.

Neuropsychiatric test

Reaction time tests have been performed in sub-populations (5) (6). Since 2011, neurocognitive testing has been done with a computerized cognitive test battery (Cogstate, Melbourne, Australia) that has been validated for HIV-infected individuals (7, 8). Four different tests from the Cogstate Brief Battery were used to assess five cognitive domains: the Detection Test measured psychomotor function and attention, the Identification test assessed speed of information processing and attention, the One Card Learning test evaluated learning, and the One Back memory test assessed working memory. (9)

Statistical methods

In most studies Wilcoxon signed rank test was used to compare the variables before and after treatment. Differences between groups were assessed with the Mann-Whitney U test.

Collaboration

Batches of CSF and serum samples are stored in -70°C in PLWH with clinical and laboratory data described above, and will be available for potential collaborators studying biomarkers during HIV infection.

For peer review only

RESULTS

A general finding resulting from our 37-year study is that HIV-1 infection in the CNS, as mirrored by CSF findings, appears early during the infectious course of the disease, and progresses slowly in the vast majority of untreated PLWH. (10, 11) Combination ART has been very effective in reducing CSF HIV-1 loads, inflammation, and markers of neural damage. (12, 13). However, minor CSF signs of long-term sequela or residual inflammatory activity have been observed during follow-up. (14-17).

When several CSF specimens from the same individual were studied, HIV-1 could be isolated from 80% of them and detected by PCR in 90% of cases, (10) with higher HIV-RNA cutoff levels required in CSF than in blood to predict positive HIV-1 isolation. (18) The viral load was approximately (mean) 1 log lower in CSF than in blood, (19-21) but CSF viral load exceeded plasma levels (CSF > plasma discordance) in 13%, with variations between different disease stages, ranging from 1% in primary HIV, 11% in neuroasymptomatic patients, and up to 30% in patients with HIV-associated dementia. (19, 21) HIV-1 RNA levels increased in CSF relative to time of infection. (11) Markers of immune stimulation such as neopterin and beta-2 microglobulin also increased in CSF during follow-up, indicating that HIV-1 CNS infection is progressive, even in a neurological asymptomatic stage. (22) However, CSF pleocytosis decreased in PLWH with severe immunosuppression, (15, 19, 21) probably because of T-cell deficiency.

Monotherapy ART with zidovudine, the only existing drug at the beginning of the AIDS epidemic, resulted in a 53 to 57% decrease in CSF neopterin concentrations. (23) The next drug on the market, didanosine, had no such effect. (24). Zidovudine-resistant variants in the brain developed during monotherapy. (25) It was obvious that combination treatment was

For peer review only

necessary to avoid viral resistance, and in 1996, when protease inhibitors was added to 2 nucleoside reverse transcriptase inhibitors (NRTI), a breakthrough in HIV care was achieved. Blood HIV load decreased to numbers below 50 copies/ml in most PLWH, and those with HIV-related symptoms clinically improved.

We found that ART was very effective in reducing viral load in CSF. Ultrasensitive PCR showed that highly effective combination treatment (HAART) resulted in undetectable HIV-1 RNA copies in CSF, although the virus was still detectable in plasma. (12) (26). Several ART combinations proved virologically effective in CSF, (26-29) and reduced blood-brain barrier integrity and intrathecal immunoglobulin production. (30, 31). CSF markers of axonal injury were also normalized. (13, 17, 28, 32) .

Studies of the highly effective short-term effects of ART were followed by long-term studies. We found that CSF viral loads were effectively suppressed over long periods of observation, but CSF signs of slight immune activation were still present in many PLWH after several years of suppressive treatment. (14, 16, 33)

While CNS infection is generally well controlled by systemic suppressive ART, there are exceptions when the HIV RNA load increases in the CSF despite suppression of the plasma viral load, a phenomenon referred to as asymptomatic CSF viral escape. (34) In these PLWH, CSF viral counts often reach just above 50 copies/ml without accompanying CSF pleocytosis or CSF signs of neuronal injury, measured as increased CSF concentration of Nfl. The lack of clinical symptoms, and the fact that the viral CSF increase was most often transient and reversed without changing therapy, has resulted in this condition to be interpreted as benign “CSF viral blips”, similar to plasma blips. (35) CSF escape is associated with increased CSF neopterin concentrations and may be related to the size of the

For peer review only

CNS HIV reservoir. Correspondingly, residual CSF viral loads below the limit of quantification by standard assays also correlate with the degree of CSF immune activation in PLWH receiving suppressive ART. (26, 36) This reinforces the view that intrathecal immune activation is driven by persistent virus in the CNS. Nevertheless, similar to findings during systemic infection, (37) treatment intensification does not seem to decrease the residual CSF viral load or inflammation, (38, 39) suggesting that there is no ongoing HIV replication during effective treatment.

Although it has not yet been definitely proven, data suggest that a stable permanent infection of cells in the CNS is established later than in the systemic viral reservoirs, (40, 41) which are highly concentrated in memory T-cell compartments within the first days of systemic HIV infection. (42) When examining anti-HIV antibody formation as a surrogate marker for antigen load and the size of the viral reservoir (43) in patients followed longitudinally during early HIV infection, serum anti-HIV antibodies emerged in blood by day 30 in untreated early infection, while CSF antibodies reached similar levels about 2 weeks later. (44) In addition, high antibody levels, comparable to those observed in chronically infected subjects, were reached several months later in CSF, as compared with blood. In addition, while treatment of chronic infection resulted in only small reductions in levels of anti-HIV antibodies in both CSF and serum, treatment during early infection substantially reduced CSF levels of antibodies, sometimes to levels close to those in HIV-negative controls. In contrast, antibody levels in serum were less affected, (44) altogether supporting this hypothesis. Our findings and those from other groups further support compartmentalization of HIV infection and immune activation in the CNS. (45-48)

For peer review only

The low-grade CNS immune activation found during suppressive ART may not be solely ascribed to HIV itself, since co-morbidities, co-infections, and life-style related factors can contribute, as elegantly shown in the COBRA study. (49, 50) The importance of appropriate controls was also demonstrated in our HIV-negative PrEP controls in whom immune activation markers and signs of neuronal injury increased as compared to non-PrEP controls. (51)

Occasionally, CSF viral escape, i.e, viral load in CSF but not in blood, is accompanied by HIV-induced neurological and neurocognitive signs and symptoms, which have been defined as "symptomatic CSF escape". (52-54) Another phenomenon that causes increased CSF viral load in well-treated PLWH is a concomitant infection in the nervous system. As an example, herpes zoster sometimes results in an inflammatory CSF reaction with slight pleocytosis, increased CSF neopterin concentrations, and increased CSF viral load, but with no detectable plasma virus. This phenomenon has been called "secondary CSF escape" and may be the result of latent virus released or detected from activated monocytes. (55)

DISCUSSION

HIV-1 infects many compartments in the human body, including the CNS. CSF surrounds the brain and is a fluid accessible to LP, which can give valuable information on infectious activity and pathological processes in the CNS. As noted earlier, in our clinical cohort most participants were asymptomatic. It can be a challenge to enroll PLWH to do repeated LP. For this reason, most cohorts studying CSF only include participants with neurological and cognitive complications, or opportunistic CNS infections having limited follow-up. To survey the whole panorama of the infectious course, we have a long-lasting collaboration with other centers, the most important being cohorts at UCSF, San Francisco, CA, USA, and Milan

For peer review only

Italy. This enables us to compare our cohort with a large number of PLWH suffering from AIDS dementia complex and other CNS complications.

HIV infection changed dramatically in severity since we began this study in 1985. At that time, we had no idea that a combination of antiretroviral drugs could have such impact. The natural course of the infection was observed with considerable data showing progressive CNS disease in several PLWH. When ART was introduced, the longitudinal project changed to monitor whether CSF viral load, markers of inflammation, and CNS injury became normalized. Some early medication with monotherapy had a limited short-term effect. It was not until 1996 when highly active antiretroviral treatment (HAART) with 3 drugs begun that the disease became a chronic latent infection with a long-life expectancy and a high quality of life. However, PLWH must remain life-long on ART. Luckily, up to now there are many modern drugs to choose from with no or minor adverse events.

There has been a debate over whether the most frequently used criteria for cognitive impairment in people with HIV, namely the Frascati criteria developed in 2007 (56) overestimate cognitive impairment. New criteria that are more appropriate for the modern era have been sought. (57, 58) The significance of mild forms of neurocognitive disorders and asymptomatic cognitive impairment detected in well-treated PLWH are controversial. Furthermore, if this condition exist, are they progressive or reversible?(2, 59). Increased CSF inflammation has been reported in PLWH on suppressive ART who experience mild cognitive impairment, (60) but the implications of this are yet to be settled. The role of aging, underlying diseases, and life-style factors of mild neurocognitive disorder are largely unknown. In such patients, longitudinal CSF studies are helpful to determine pathogenic factors that may affect the CNS.

For peer review only

Despite our intention to follow participants annually, it was difficult to accomplish. Some participants consented to repeated LP, but at longer intervals than annually. Moreover, the Covid-19 pandemic halted several clinical studies for two years. Another limitation is that our cohort included relatively few PLWH with advanced disease and CNS complications. By means of international collaborations, more CSF data from patients with severe neurological complications and opportunistic CNS infections have been used for comparison with our cohort data in several cross-sectional studies. A limitation is also the infrequent use of extensive neuropsychiatric test batteries. Several attempts to include regular neuropsychiatric tests in the protocol failed due to methodological difficulties and a lack of resources. Furthermore, it was difficult to enroll a valid control group. Another complication while managing neuropsychiatric analyses in longitudinal studies involving repeated tests is the learning factor, which may result in false test results. In addition, there were many participants with language difficulties.

A major strength of our study is its uniquely long follow-up time, with CSF data from a population of PLWH with a predominately neuroasymptomatic clinical appearance, which we believe has never been done before. In addition, the study was performed with a very strict protocol for collecting and storing CSF/blood samples at one center, and only engaging a handful of clinicians to enhance consistency and uniformity.

Our future objectives involve several questions. Are there any active, ongoing inflammatory or neurotoxic processes remaining in the brain, despite successful virological treatment? Are there any complications from the CNS caused by chronic antiretroviral medication? Characterization of the CNS HIV reservoir and its establishment is still largely unknown.

What is the importance of compartmentalized CNS infection, and if peripheral eradication treatment in the blood and the lymphatic system proves successful in the future, is it possible bring about the same effect in the CNS? The study will continue with follow-up of already included participants and recruitment of newly diagnosed PLWH.

Ethics

At the time of HIV diagnosis, each person being diagnosed is asked for consent to be included in the study, and to decline without stating any reasons. Participants always have the right to exit the study. They may also request an extract of their data from the register, at no charge, in accordance with the European General Data Protection Regulation (GDPR 2016/679) and the Swedish Data Protection Act (2018:218).

Biobank: Sahlgrenska University Hospital, Gothenburg, Sweden, no. 890

International collaborations: Collaborations with HIV cohorts from other countries: University of California (UCSF); Prof. Richard W Price; University of Milano, Italy; Prof. Paola Cinque; University of Sidney, Australia; Prof. Bruce Brew; University of Innsbruck, Austria, Prof. Dietmar Fuchs; University of North Carolina (UNC), Chapel Hill; Prof. Ron Swanstrom.

Authors contribution: LH and MG designed the study, examined and collected CSF from the majority of the participants, registered the results, and wrote the paper with equal contribution.

For peer review only

Funding to report for this paper: This work was supported by the Swedish state under an agreement between the Swedish government and the county councils (ALF agreement ALFGBG-965885) and the Swedish Research Council (2021-06545)

Competing interests: LH has no competing interest. MG has received research grants from Gilead Sciences and Janssen-Cilag, and honoraria as a speaker and/or scientific advisor from Amgen, AstraZeneca, Biogen, Bristol-Myers Squibb, Gilead Sciences, GlaxoSmithKline/ViiV, Janssen-Cilag, MSD, Novocure, Novo Nordic, Pfizer and Sanofi.

Patient consent for publication: Not required

Ethics approval: This cohort study has been approved by the Swedish Ethics Review Authority.

Provenance and peer review: Not commissioned; externally peer reviewed.

Data availability statement: Data can be made available upon legitimate request pending ethical approval.

For peer review only

References

1. Dore GJ, Correll PK, Li Y, et al. Changes to AIDS dementia complex in the era of highly active antiretroviral therapy. *AIDS* 1999;13(10):1249–53.

2. McArthur JC, Brew BJ, Nath A. Neurological complications of HIV infection. *Lancet Neurol* 2005;4(9):543–55.

3. Hagberg L, Malmvall BE, Svennerholm L, et al. Guillain-Barre syndrome as an early manifestation of HIV central nervous system infection. *Scand J Infect Dis* 1986;18(6):591–2.

4. Chiodi F, Asjo B, Fenyo EM, et al. Isolation of human immunodeficiency virus from cerebrospinal fluid of antibody-positive virus carrier without neurological symptoms. *Lancet* 1986;2(8518):1276–7.

5. Dunlop O, Bjorklund RA, Abdelnoor M, et al. Five different tests of reaction time evaluated in HIV seropositive men. *Acta Neurol Scand* 1992;86(3):260–6.

6. Mellgren A, Norkrans G, Hagberg L, et al. Slowed reaction time in HIV-1-infected patients without AIDS. *Acta Neurol Scand* 2000;102(3):169–74.

7. Cysique LA, Maruff P, Darby D, et al. The assessment of cognitive function in advanced HIV-1 infection and AIDS dementia complex using a new computerised cognitive test battery. A*rch Clin Neuropsychol* 2006;21(2):185–94.

8. Maruff P, Thomas E, Cysique L, et al. Validity of the CogState brief battery: relationship to standardized tests and sensitivity to cognitive impairment in mild traumatic brain injury, schizophrenia, and AIDS dementia complex. *Arch Clin Neuropsychol* 2009;24(2):165–78.

9. Yilmaz A, Mellgren A, Fuchs D, et al. Switching from a regimen containing abacavir/lamivudine or emtricitabine/tenofovir disoproxil fumarate to emtricitabine/tenofovir alafenamide fumarate does not affect central nervous system HIV-1 infection. *Infect Dis (Lond)* 2019;51(11–12):838–46.

For peer review only

10. Chiodi F, Keys B, Albert J, et al. Human immunodeficiency virus type 1 is present in the cerebrospinal fluid of a majority of infected individuals. *J Clin Microbiol* 1992;30(7):1768–71.

11. Gisslen M, Hagberg L, Fuchs D, et al. Cerebrospinal fluid viral load in HIV-1-infected patients without antiretroviral treatment: a longitudinal study. *J Acquir Immune Defic Syndr Hum Retrovirol* 1998;17(4):291–5.

12. Gisslen M, Norkrans G, Svennerholm B, et al. HIV-1 RNA detectable with ultrasensitive quantitative polymerase chain reaction in plasma but not in cerebrospinal fluid during combination treatment with zidovudine, lamivudine and indinavir. *AIDS* 1998;12(1):114–6.

13. Abdulle S, Mellgren A, Brew BJ, et al. CSF neurofilament protein (NFL)—a marker of active HIV-related neurodegeneration. *J Neurol* 2007;254(8):1026–32.

14. Eden A, Price RW, Spudich S, et al. Immune activation of the central nervous system is still present after > 4 years of effective highly active antiretroviral therapy. *J Infect Dis* 2007;196(12):1779–83.

15. Hagberg L, Forsman A, Norkrans G, et al. Cytological and immunoglobulin findings in cerebrospinal fluid of symptomatic and asymptomatic human immunodeficiency virus (HIV) seropositive patients. *Infection* 1988;16(1):13–8.

16. Ulfhammer G, Eden A, Mellgren A, et al. Persistent central nervous system immune activation following more than 10 years of effective HIV antiretroviral treatment. *AIDS* 2018;32(15):2171–8.

17. Jessen Krut J, Mellberg T, Price RW, et al. Biomarker evidence of axonal injury in neuroasymptomatic HIV-1 patients. *PLoS One* 2014;9(2):e88591.

18. Andersson LM, Svennerholm B, Hagberg L, et al. Higher HIV-1 RNA cutoff level required in cerebrospinal fluid than in blood to predict positive HIV-1 isolation. *J Med Virol* 2000;62(1):9–13.

For peer review only

19. Gisslen M, Fuchs D, Svennerholm B, et al. Cerebrospinal fluid viral load, intrathecal immunoactivation, and cerebrospinal fluid monocytic cell count in HIV-1 infection. *J Acquir Immune Defic Syndr* 1999;21(4):271–6.

20. Spudich SS, Nilsson AC, Lollo ND, et al. Cerebrospinal fluid HIV infection and pleocytosis: relation to systemic infection and antiretroviral treatment. *BMC Infect Dis* 2005;5:98.

21. Ulfhammer G, Eden A, Antinori A, et al. Cerebrospinal fluid viral load across the spectrum of untreated HIV-1 infection: a cross-sectional multi-center study. *Clin Infect Dis* 2022;75(3):493–502.

22. Gisslen M, Chiodi F, Fuchs D, et al. Markers of immune stimulation in the cerebrospinal fluid during HIV infection: a longitudinal study. *Scand J Infect Dis* 1994;26(5):523–33.

23. Hagberg L, Norkrans G, Andersson M, et al. Cerebrospinal fluid neopterin and beta 2-microglobulin levels in neurologically asymptomatic HIV-infected patients before and after initiation of zidovudine treatment. *Infection* 1992;20(6):313–5.

24. Hagberg L, Norkrans G, Gisslen M, et al. Intrathecal immunoactivation in patients with HIV-1 infection is reduced by zidovudine but not by didanosine. *Scand J Infect Dis* 1996;28(4):329–33.

25. Di Stefano M, Norkrans G, Chiodi F, et al. Zidovudine-resistant variants of HIV-1 in brain. *Lancet* 1993;342(8875):865.

26. Yilmaz A, Svennerholm B, Hagberg L, et al. Cerebrospinal fluid viral loads reach less than 2 copies/ml in HIV-1-infected patients with effective antiretroviral therapy. *Antivir Ther* 2006;11(7):833–7.

27. Gisslen M, Svennerholm B, Fuchs D, et al. Neurological efficacy of stavudine, zidovudine, and lamivudine. *Lancet* 1998;352(9125):402–3.

For peer review only

28. Mellgren A, Price RW, Hagberg L, et al. Antiretroviral treatment reduces increased CSF neurofilament protein (NFL) in HIV-1 infection. *Neurology* 2007;69(15):1536–41.

29. Yilmaz A, Stahle L, Hagberg L, et al. Cerebrospinal fluid and plasma HIV-1 RNA levels and lopinavir concentrations following lopinavir/ritonavir regimen. *Scand J Infect Dis* 2004;36(11–12):823–8.

30. Abdulle S, Hagberg L, Gisslen M. Effects of antiretroviral treatment on blood-brain barrier integrity and intrathecal immunoglobulin production in neuroasymptomatic HIV-1-infected patients. *HIV Med* 2005;6(3):164–9.

31. Anesten B, Zetterberg H, Nilsson S, et al. Effect of antiretroviral treatment on blood-brain barrier integrity in HIV-1 infection. *BMC Neurol* 2021;21(1):494.

32. Yilmaz A, Blennow K, Hagberg L, et al. Neurofilament light chain protein as a marker of neuronal injury: review of its use in HIV-1 infection and reference values for HIV-negative controls. *Expert Rev Mol Diagn* 2017;17(8):761–70.

33. Yilmaz A, Yiannoutsos CT, Fuchs D, et al. Cerebrospinal fluid neopterin decay characteristics after initiation of antiretroviral therapy. *J Neuroinflammation* 2013;10:62.

34. Eden A, Fuchs D, Hagberg L, et al. HIV-1 viral escape in cerebrospinal fluid of subjects on suppressive antiretroviral treatment. *J Infect Dis* 2010;202(12):1819–25.

35. Eden A, Nilsson S, Hagberg L, et al. Asymptomatic cerebrospinal fluid HIV-1 viral blips and viral escape during antiretroviral therapy: a longitudinal study. *J Infect Dis* 2016;214(12):1822–5.

36. Dahl V, Peterson J, Fuchs D, et al. Low levels of HIV-1 RNA detected in the cerebrospinal fluid after up to 10 years of suppressive therapy are associated with local immune activation. *AIDS* 2014;28(15):2251–8.

For peer review only

37. Dinoso JB, Kim SY, Wiegand AM, et al. Treatment intensification does not reduce residual HIV-1 viremia in patients on highly active antiretroviral therapy. *Proc Natl Acad Sci USA* 2009;106(23):9403–8.

38. Dahl V, Lee E, Peterson J, et al. Raltegravir treatment intensification does not alter cerebrospinal fluid HIV-1 infection or immunoactivation in subjects on suppressive therapy. *J Infect Dis* 2011;204(12):1936–45.

39. Yilmaz A, Verhofstede C, D'Avolio A, et al. Treatment intensification has no effect on the HIV-1 central nervous system infection in patients on suppressive antiretroviral therapy. *J Acquir Immune Defic Syndr* 2010;55(5):590–6.

40. Hellmuth J, Slike BM, Sacdalan C, et al. Very early initiation of antiretroviral therapy during acute HIV infection is associated with normalized levels of immune activation markers in cerebrospinal fluid but not in plasma. *J Infect Dis* 2019;220(12):1885–91.

41. Gisslen M, Hunt PW. Antiretroviral treatment of acute HIV infection normalizes levels of cerebrospinal fluid markers of central nervous system (CNS) inflammation: a consequence of a reduced CNS reservoir? *J Infect Dis* 2019;220(12):1867–9.

42. Hunt PW, Lee SA, Siedner MJ. Immunologic biomarkers, morbidity, and mortality in treated HIV infection. *J Infect Dis* 2016;214 Suppl 2:S44–50.

43. Lee SA, Bacchetti P, Chomont N, et al. Anti-HIV antibody responses and the HIV reservoir size during antiretroviral therapy. *PLoS One* 2016;11(8):e0160192.

44. Burbelo PD, Price RW, Hagberg L, et al. Anti-human immunodeficiency virus antibodies in the cerebrospinal fluid: evidence of early treatment impact on central nervous system reservoir? *J Infect Dis* 2018;217(7):1024–32.

45. Bednar MM, Sturdevant CB, Tompkins LA, et al. Compartmentalization, viral evolution, and viral latency of HIV in the CNS. *Curr HIV/AIDS Rep* 2015;12(2):262–71.

For peer review only

46. Joseph SB, Kincer LP, Bowman NM, et al. Human immunodeficiency virus type 1 RNA detected in the central nervous system (CNS) after years of suppressive antiretroviral therapy can originate from a replicating CNS reservoir or clonally expanded cells. *Clin Infect Dis* 2019;69(8):1345–52.

47. Gisslen M, Svennerholm B, Norkrans G, et al. Cerebrospinal fluid and plasma viral load in HIV-1-infected patients with various anti-retroviral treatment regimens. *Scand J Infect Dis* 2000;32(4):365–9.

48. Gisslen M, Keating SM, Spudich S, et al. Compartmentalization of cerebrospinal fluid inflammation across the spectrum of untreated HIV-1 infection, central nervous system injury and viral suppression. *PLoS One* 2021;16(5):e0250987.

49. De Francesco D, Wit FW, Burkle A, et al. Do people living with HIV experience greater age advancement than their HIV-negative counterparts? *AIDS* 2019;33(2):259–68.

50. Booiman T, Wit FW, Maurer I, et al. High cellular monocyte activation in people living with human immunodeficiency virus on combination antiretroviral therapy and lifestyle-matched controls is associated with greater inflammation in cerebrospinal fluid. *Open Forum Infect Dis* 2017;4(3):ofx108.

51. Robertson J, Eden A, Nystrom K, et al. Increased immune activation and signs of neuronal injury in HIV-negative people on preexposure prophylaxis. *AIDS* 2021;35(13):2129–36.

52. Canestri A, Lescure FX, Jaureguiberry S, et al. Discordance between cerebral spinal fluid and plasma HIV replication in patients with neurological symptoms who are receiving suppressive antiretroviral therapy. *Clin Infect Dis* 2010;50(5):773–8.

53. Peluso MJ, Ferretti F, Peterson J, et al. Cerebrospinal fluid HIV escape associated with progressive neurologic dysfunction in patients on antiretroviral therapy with well controlled plasma viral load. *AIDS* 2012;26(14):1765–74.

For peer review only

54. Ferretti F, Gisslen M, Cinque P, et al. Cerebrospinal fluid HIV escape from antiretroviral therapy. *Curr HIV/AIDS Rep* 2015;12(2):280–8.

55. Hagberg L, Price RW, Zetterberg H, et al. Herpes zoster in HIV-1 infection: the role of CSF pleocytosis in secondary CSF escape and discordance. *PLoS One.* 2020;15(7):e0236162.

56. Antinori A, Arendt G, Becker JT, et al. Updated research nosology for HIV-associated neurocognitive disorders. *Neurology* 2007;69(18):1789–99.

57. Gisslen M, Price RW, Nilsson S. The definition of HIV-associated neurocognitive disorders: are we overestimating the real prevalence? *BMC Infect Dis* 2011;11:356.

58. Nightingale S, Dreyer AJ, Saylor D, et al. Moving on from HAND: why we need new criteria for cognitive impairment in persons living with human immunodeficiency virus and a proposed way forward. *Clin Infect Dis* 2021;73(6):1113–8.

59. Robertson KR, Smurzynski M, Parsons TD, et al. The prevalence and incidence of neurocognitive impairment in the HAART era. *AIDS* 2007;21(14):1915–21.

60. Eden A, Marcotte TD, Heaton RK, et al. Increased intrathecal immune activation in virally suppressed HIV-1 infected patients with neurocognitive impairment. *PLoS One* 2016;11(6):e0157160.

Figure 1. Flow chart of 662 people living with HIV (PLWH) included in the Gothenburg HIV CSF study (1985 to Apr 7, 2022).

For peer review only

Figure 1

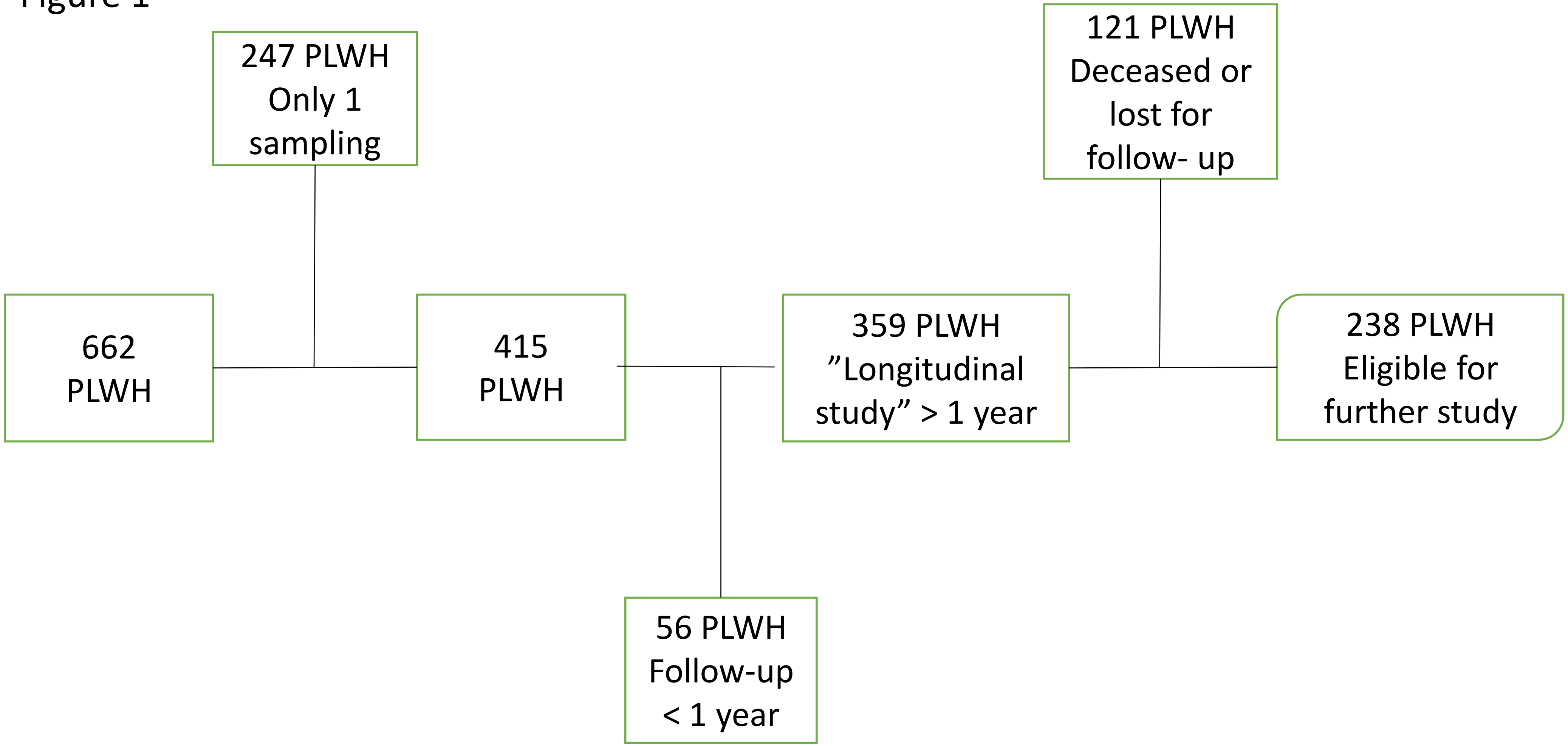
247 PLWH Only 1 sampling
121 PLWH Deceased or lost for follow- up
662 PLWH
415 PLWH
359 PLWH ”Longitudinal study” > 1 year
238 PLWH Eligible for further study
56 PLWH Follow-up < 1 year
